# Patient Expectations of Assigned Treatments Impact Strength of Randomised Control Trials

**DOI:** 10.3389/fmed.2021.648403

**Published:** 2021-06-17

**Authors:** Roberto Truzoli, Phil Reed, Lisa A. Osborne

**Affiliations:** ^1^Department of Biomedical and Clinical Sciences “L. Sacco”, University of Milan, Milan, Italy; ^2^Department of Psychology, Swansea University, Swansea, United Kingdom; ^3^School of Psychology and Counselling, The Open University, Milton Keynes, United Kingdom; ^4^Women's Health, Swansea Bay University Health Board, Swansea, United Kingdom

**Keywords:** RCT, clinical outcome-effectiveness, patient expectations, patient variables, false negatives, Monte Carlo simulations

## Abstract

Patient engagement with treatments potentially poses problems for interpreting the results and meaning of Randomised Control Trials (RCTs). If patients are assigned to treatments that do, or do not, match their expectations, and this impacts their motivation to engage with that treatment, it will affect the distribution of outcomes. In turn, this will impact the obtained power and error rates of RCTs. Simple Monto Carlo simulations demonstrate that these patient variables affect sample variance, and sample kurtosis. These effects reduce the power of RCTs, and may lead to false negatives, even when the randomisation process works, and equally distributes those with positive and negative views about a treatment to a trial arm.

## Introduction

The Randomised Control Trial (RCT) is a flexible tool, capable of addressing many questions in clinical settings, grounded on the notion that outcomes have not been differentially determined by factors other than allocated treatment. Based on this assumption, RCTs allow causal evidence that a treatment leads to an improvement, and this improvement cannot be attributed to any characteristic other than the treatment. Consequently, RCTs remain the “gold standard” for assessing treatment efficacy, and great weight is given to their findings in informing official practise guidelines.

Nevertheless, debate about the use and value of RCTs in clinical practise has been vibrant and ongoing across many different healthcare fields. In particular, the notion that patient-characteristics are not of “first line” importance in assessing outcomes jars with contemporary conjoint decision-making practises in health settings. Recognising the patient as an active collaborator in their treatment (co-production) has implications for how this research strategy should be viewed. Moreover, it is apparent that patients” psychological characteristics, including how much they engage with treatments, impact outcomes. The present perspective-article suggests that views around the usefulness of RCTs, within many clinical settings, may benefit from consideration of issues related to patient psychological characteristics.

Patient expectations may influence results, as randomisation to groups perceived as less efficacious may increase attrition. However, effects of patient-characteristics on RCT outcomes may go beyond attrition. Patient expectations and motivation may determine aspects of sample statistics and trial results in ways that cannot be accommodated by RCTs. In particular, they may reduce statistical power relating to patients who complete the trial. To illustrate debates about the usefulness of RCTs, examples from the field of Physiotherapy will be used, as patient engagement with treatment is a key part of treatment effectiveness. A Monte Carlo simulation will illustrate the possible impacts of patient psychological characteristics on the distribution of data from an RCT.

## RCTs in Physiotherapy

There is little doubt that, within Physiotherapy, there is an increasing trend toward usage of RCTs in assessing treatment outcome-effectiveness (1, 2). Several reviews have noted an increase in numbers of published RCT studies, over the last 40 years. Analysing data contained in the Physiotherapy Evidence Database [PEDro; (3)], Kamper et al. (1) noted about 24,000 RCTs had been published by 2014, compared to under 1,000 by 1980. A similar pattern was observed by Kelly et al. (2), when examining articles published in the journal, *Physiotherapy*.

**Figure 1:**
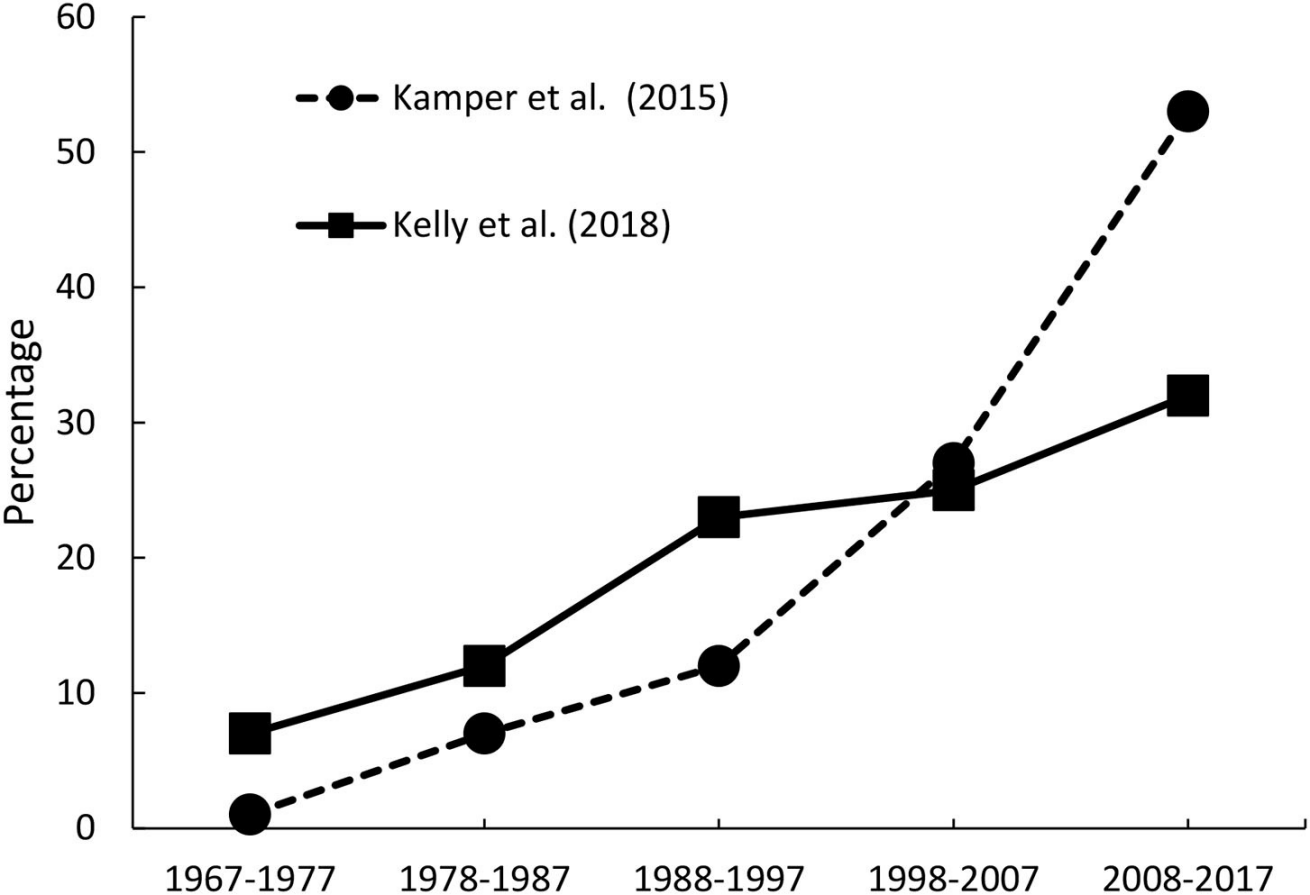
Percentage of RCT studies, published across the past 40 years, falling into each successive 10-year period, reported by Kamper et al. (1) and Kelly et al. (2).

Regardless of the absolute numbers, what is clear is that RCTs are an increasing part of the evidence-base for Physiotherapy. Figure 1 presents the percentage of RCT studies, published across the past 40 years, falling into each successive 10-year period, reported by Kamper et al. (1) and Kelly et al. (2). As the two reviews employed different databases, with different totals of studies, these figures are displayed as percentages. Despite some differences, the trend toward increasing use of RCTs is clear in both reports. Whether this trend reflects an increasing acknowledgement that RCTs are the “gold standard,” and should be adopted within Physiotherapy as a matter of course (2), an increasing realisation that RCTs can be conducted well in Physiotherapy (1), or an increasing recognition of the reality that RCTs are important from policy-guidance panels, is all debateable.

## Assessing RCT Quality

Conducting an RCT is one thing; the RCT being of good quality is another. If the objective is to impact clinical practise, then the RCT needs to be of sufficient quality to be considered by policy-guideline boards, like the Cochrane Database. The introduction of the PEDro scale, to rate methodological rigour of RCTs conducted in Physiotherapy, has been credited with helping to improve their quality (3, 4). This scale includes items describing various aspects of study design, which can be used to pinpoint problems with a study, or summed to give an overall RCT-quality score (5). According to a review conducted by Moseley et al. (6), using the PEDro mean overall score, the quality of RCTs in Physiotherapy has increased by about 0.6 points per decade since 1960. Armijo-Olivo et al. (7) suggest that about 60% of Physiotherapy RCTs evaluated were of an adequate quality, according to the PEDro. Thus, focus on methodological rigour of RCTs has served to improve their quality, which should be beneficial for clinical practise.

Of key importance is the impact that RCT studies have on practise and policy, and inclusion in Cochrane Reviews, and/or NICE Guidelines, are key indices of potential impact. The major problems with RCTs conducted in Physiotherapy, which mirror those conducted in Clinical Psychology, are lacks of both random sequence generation, and of concealed or blind assignment, as suggested by the “Cochrane Risk of Bias” tool. Lack of randomisation (or lack of detailed descriptions of the process), lack of double-blinding of those delivering and receiving interventions, and high attrition-rates which can be differential across groups, are aspects that most often undermine RCT quality. The three-item Jadad Scale has been used to assess these aspects of RCTs (8). However, even if these issues could be addressed, it would not mean that RCTs are always appropriate devices to assess clinical outcomes. This debate has been aired to a large extent in Physiotherapy, and it resonates with concerns from Psychology applied to clinical settings.

## Critiques of RCTs

The use of RCTs has not gone unchallenged in many healthcare fields, and a number of articles have suggested that there may be more appropriate approaches for Physiotherapy (9, 10), and healthcare sciences in general (11, 12). While there is little doubt of the commitment of most clinicians to evidence-based medicine, this does not automatically mean a commitment to RCTs (9). In fact, other approaches to evidence-collection are widely used, and, sometimes, considered more appropriate (13). Indeed, good-quality controlled studies, or prospective observational studies, can provide better evidence than low-quality RCTs, according to GRADE recommendations (14). Djulbegovic and Guyatt (15) noted that overpowered RCTs, with too strict inclusion criteria, and which include comparisons with “no treatment” (very rarely a clinical option), will not give evidence of any great practical value. Additionally, over-focus on RCTs can relegate the “voice of clinical experience” down the evidence-hierarchy (9); evidence-based medicine requires that RCTs inform, *but do not replace*, clinical judgement (16).

In relation to Physiotherapy, Bithell (9) summarised many objections to RCTs, relevant to any setting where patients play active and co-productive roles in their treatments. The key objections being: RCTs are time-consuming and expensive to conduct, making them unlikely to impact clinical practise; it is difficult to gain access to appropriate populations; and pre-specified, invariable treatments are simply not appropriate, where treatments have to adapt to individual patients. All of these points are equally-applicable arguments within Clinical Psychology—especially the final point. Clinically, what is important is the manner in which an *individual* patient, not the *average* patient, responds (13, 17).

Krauss (12) noted many potential areas of bias, that are extremely difficult to overcome using the RCT-method, and which apply equally in Physiotherapy and Clinical Psychology. One in particular is highly relevant—that is, the patient tends to change over time, in many ways. A fact that runs counter to the “background-traits-remain-constant” assumption inherent in RCTs. At the end of a trial, these influences can impact outcomes, and bias results, just as much as at the beginning of a trial, but these influences have not necessarily been randomised-out; the longer the trial, the greater the likelihood of a biassing impact on the outcome that these patient-changes will have.

The interaction between the intervention and the patient is at the core of a critique of RCTs within Psychology (11). As noted for Physiotherapy, treatments vary from session-to-session, and often within sessions. This variation is not easy to manualise, in anything other than a vague way. Moreover, the particular therapy technique adopted is not the sole, nor perhaps critical, variable producing treatment outcomes. Specifying the treatment does not help understand what has gone on within therapy sessions, as developing rapport with a patient, and patient involvement, are also critical but nebulous (18).

In all of this debate, of central concern is the assumption of causal-direction—the suggestion that the treatment produces patient change. However, this assumes that patients are passive recipients, and that their characteristics are, at best, an inconvenience to the treatment (certainly to the RCT). Moreover, if the independent variable (the treatment) interacts with the dependent variable (the patient's behaviour), that is, the therapy changes as the patient's behaviour changes, then the rationale for the RCT is undermined. It is often assumed that random assignment of patients to treatments reduces the impact of such factors (19). However, as highlighted in the next sections, random assignment might exacerbate this issue.

## Patient Characteristics

It is increasingly apparent in clinical practise that psychological characteristics of patients, including how much they engage with treatments, impact outcomes (20). A consideration reflected in increased usage of patient motivational-supports (21). Coupled with including patients as active contributors to treatment-decisions, this consideration poses potential statistical problems for interpreting RCT results.

If a patient is assigned to a treatment that does, or does not, match their expectations, this could impact their motivation to engage with that treatment, subsequently affecting their treatment outcomes. This uncontrolled (and uncontrollable) process may impact the obtained power and error rates of RCTs, in ways that have not yet been thoroughly considered. Such impacts on the ability to interpret statistical outcomes could emerge through a number of routes, including effects on: group differences, sample variances, and sample kurtoses—each with statistical effects, and consequences, for RCTs, not yet fully determined.

### Simulations

To assess potential impacts of random assignment of individuals to groups, which may or may not match their expectations, Monto Carlo simulations, using 1,000 repetitions of each scenario, determined effects on sample mean, standard deviation, skewness, and kurtosis. For simplicity, it was assumed that an intervention, without influence of patient motivation-to-engage, would produce a normally distributed range of outcomes, with a mean of 100, and a standard deviation of 15. It was also assumed that patients could take one of three views of their treatment: positive (adding to treatment effect); neutral (not altering treatment effect); and negative (reducing treatment effect).

**Figure 2:**
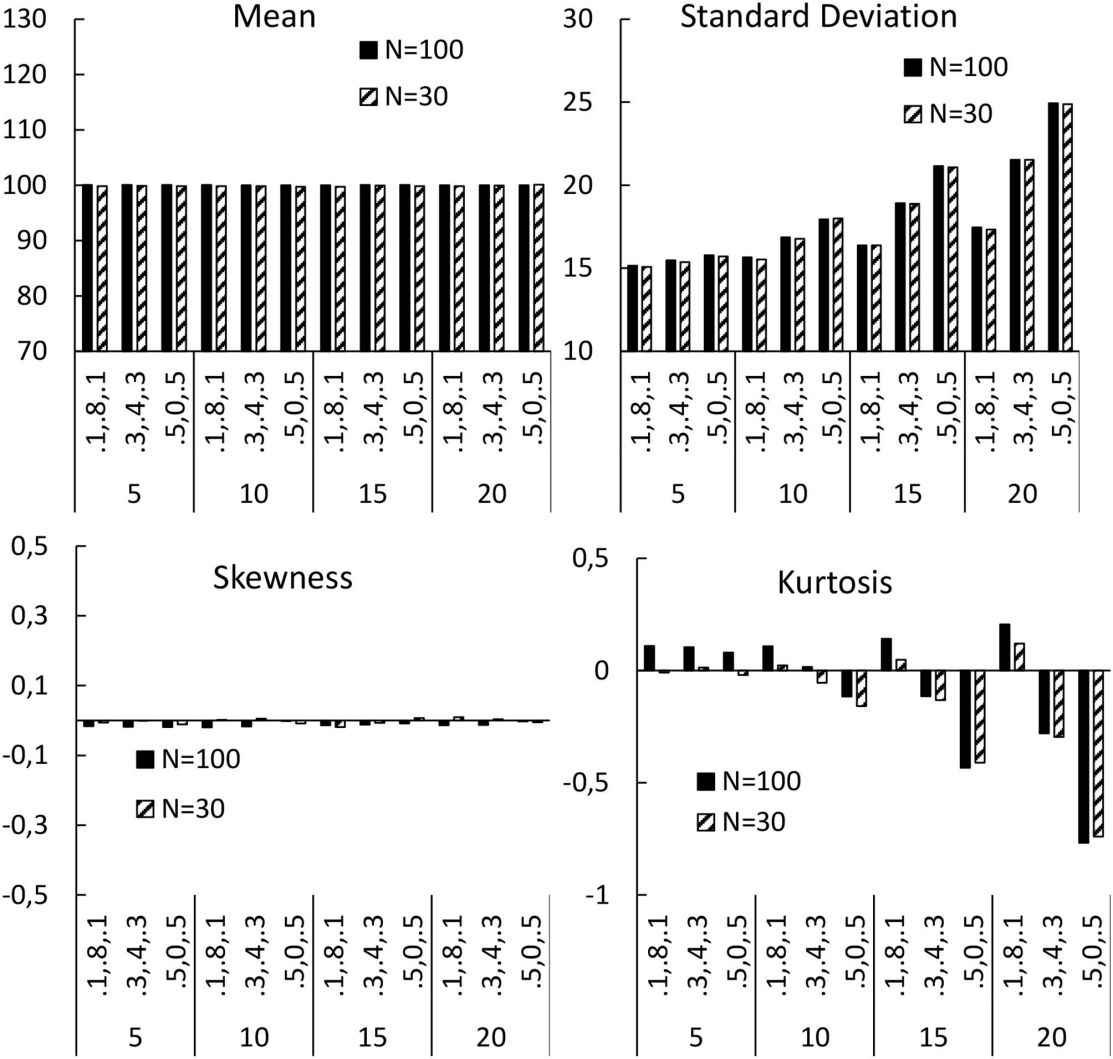
Means for the sample mean, standard deviation, skewness, and kurtosis, calculated under each condition. Bottom axes are the effect of motivation (5, 10, 15, 20), and the proportion of the sample taking a particular attitude to a treatment (0.1, 0.8, 0.1 = 10% negative, 80% neutral, 10% positive; 0.3, 0.4, 0.3 = 30% negative, 40% neutral, 30% positive; 0.5,0, 0.5 = 50% negative, 0% neutral, 50% positive).

A number of simulations were conducted involving the effects of: larger (*N* = 100) and smaller (*N* = 30) samples; a range of impacts of motivation on outcomes (adding or subtracting 5, 10, 15, or 20 points to outcomes); and a range of proportions of individuals adopting a particular stance toward a treatment (10% positive/negative; 30% positive/negative; 50% positive/negative). The means for the sample mean, standard deviation, skewness, and kurtosis, were calculated under each condition (shown in Figure 2).

Inspection of Figure 2 shows that two of the parameters were affected by patients' orientations toward the treatment: the standard deviation, and the kurtosis; while the mean, and the skewness, were not impacted. The effects grew larger as the impact of the orientation increased, and were most noticeable when the impact was to add/subtract 15, or more, points to/from the outcome (i.e., one standard deviation, or more). The effect was also more pronounced, the greater the proportion of the sample who were taken to adopt a particular stance toward the treatment. These effects occurred for both small and large samples, even with the randomisation being successful—that is, equal numbers of patients taking positive and negative stances toward the treatment. Thus, RCTs cannot overcome this problem, and failing to account for patient preferences, by randomly assigning patients to treatments, may actually induce this problem.

### Impacts on Sample Variance

This effect is important, as the power of a statistical test is negatively related to the variance. If some patients increase the clinical outcome of the treatment (due to the match of their assigned treatment with their expectations), and some patients reduce their improvement (due to a mismatch), then the variance in the sample is increased. As variance is inversely related to power, this could reduce power for an RCT, making it harder to reject the null hypothesis, and leading to more Type-2 errors. Building on the current simulation, assuming an intervention-group mean of 107.5, a control mean of 100, and standard deviation (SD) of 15 (medium effect size), 94% power is achieved with 100 participants per group, and 49% with 30 per group. With an SD of 21, there is 71% power for 100 participants, and 28% for 30 per group. When SD = 30, power drops to 42% for 100 per group, and 16% for 30 per group. The pronounced reduction may account for the relative difficulty in establishing a statistically significant effect of a treatment when an RCT is adopted, even when it is regularly seen in controlled studies.

### Impacts on Sample Distribution

The impact of patients taking positive or negative orientations toward a treatment was to make the kurtosis negative; implying a “flatter” (platykurtic) distribution of scores, with more data in the “tails.” Although some extreme scorers are affected by their expectations of treatment, it is necessarily the case that more patients from the centre of the distribution will move away from that centre to the extremes. Deviations in kurtosis make parametric statistical tests less reliable, and the power of a statistical test reduces as the excess kurtosis increases (22), making Type-2 errors more likely. As more data from two groups gather in their distribution “tails,” there is more overlap, which reduces chances of statistical significance. In fact, avoiding *t*-tests has been recommended when there is such low kurtosis, due to low power (23). An alternative might be to conduct non-parametric tests, but these statistical procedures, on the whole, are more conservative, and less powerful, than their parametric alternatives, and this would not really solve the power/Type-2 error problem.

## Implications

The effects of patient expectations and motivations on RCTs opens new questions about the validity of these trials when patent engagement is integral to treatment. The focus of this analysis was face-to-face Physiotherapy and Psychotherapy. However, similar considerations apply to pharmacological, and to computerised physical or psychological, trials.

Patients randomised to treatments perceived as less efficacious may prematurely terminate studies. Such attrition is a critical consideration, and measuring compliance is essential. In drug trials, pharmacokinetics (PK) and pharmacodynamics (PD) assess compliance (24). PK characterises drug exposure through absorption, distribution, metabolism, excretion; PD describes biochemical and/or molecular interactions. However, the impacts on PK/PD of physiological responses engendered by assignment to unwanted treatment is unknown.

Assessment of compliance and engagement with Physiotherapy/Psychotherapy trials, beyond measuring attendance, are less clear—especially when “homework” is involved (21). To help assess compliance, treatment procedures need careful description in protocols. Although many interventions are individually structured (9, 10), monitoring compliance and fidelity of individualised interventions aids trial-outcome reliability (25). Moreover, increasing motivation, before and during interventions, may bolster treatment and trial power; perhaps by adopting motivational and values support training (21).

However, the current simulations imply that patient expectations impact RCTs beyond compliance, affecting sample statistics, reducing statistical power, and increasing Type-2 errors (falsely denying treatment efficacy). This is a harder issue to resolve, certainly affecting non-blinded or single-blinded trials, where patients are informed about their treatments. Double-blinded trials are considered methodologically superior, but also will be subject to these effects—patients become aware of the nature of their physical/psychological therapies, and form their opinions of these treatments, even when not explicitly informed about the natures of their interventions (10). This may also be true for pharmacological treatments where drugs have discriminable physical effects. Moreover, it is not clear how traditional physical and psychological treatments could (or should) be blinded from the professionals delivering them (11, 13).

Computerised therapies (e.g., digitised psychoeducational pelvic-floor programmes, or Cognitive Behaviour Therapy interventions) may not have to blind the delivery-agent, but the investigated phenomenon will still be important, as patient-engagement with the app is causal to clinical efficacy (26). Given this, even double-blinded studies of digital-delivery systems will be impacted by patent expectations and motivations. Perhaps the optimal procedure is to measure patient expectations/motivations to control for their effects, although this acknowledges that RCTs *per se* do not control for all problems.

## Conclusion

The simulation suggests that, whenever patients are actively engaged in their treatments, their expectations and motivations play a role in compliance, and determine the power of the trials. These considerations may be addressed in a variety of ways in different fields (e.g., physical vs. pharmacological therapies), and there are aspects of good practise that may mitigate some of these deleterious effects on RCT outcomes. However, ultimately, this may be regarded as a “brute” objection to RCTs that cannot be controlled away, and the best that may be achievable is to measure the influence, and recognise the advantages of alternative assessment-methodologies in some situations.

These considerations add to concerns over the assumption that RCTs are *the* “gold standard” for determining the outcome-effectiveness of any clinical treatment. If patient expectations and preferences are important determinants of treatment-outcomes, then the impact of these patient-variables on the statistical interpretability of RCTs is a serious concern in clinical settings. Given the increasing involvement of patients in joint decision-making about their treatments, these concerns would seem to warrant further attention being paid to how to measure and approach such effects, due to their potential adverse influences on the power and error rates of RCTs.

## Data Availability Statement

The raw data supporting the conclusions of this article will be made available by the authors, without undue reservation.

## Author Contributions

All authors listed have made a substantial, direct and intellectual contribution to the work, and approved it for publication.

## Conflict of Interest

The authors declare that the research was conducted in the absence of any commercial or financial relationships that could be construed as a potential conflict of interest.

## References

[1] Kamper SJ Moseley AM Herbert RD Maher CG Elkins MR Sherrington C. 15 years of tracking physiotherapy evidence on PEDro, where are we now? Br J Sports Med. (2015) 49:907–9. 10.1016/j.physio.2015.03.3565 25833902

[2] Kelly G Reilly A Moloney H Moran J Cunningham C Broderick J. 50 years of randomised controlled trials published in the journal Physiotherapy: a review 1967 to 2017. Physiotherapy. (2018) 104:359–66. 10.1016/j.physio.2018.08.005 30318126

[3] Blobaum PM. Physiotherapy evidence database (PEDro), (Electronic Resource Review). J Med Libr Assoc. (2006) 94: 477–8.

[4] Kamper SJ Moseley AM Herbert RD Maher CG Elkins MR Sherrington C. 15 years of tracking physiotherapy evidence on PEDro, where are we now? Br J Sports Med. (2015) 49:907–9. 10.1136/bjsports-2014-094468 25833902

[5] Maher CG Sherrington C Herbert RD. Reliability of the PEDro scale for rating quality of randomized controlled trials. Phys Ther. (2003) 83:713–21. 10.1093/ptj/83.8.713 12882612

[6] Moseley AM Herbert RD Maher CG Sherrington C Elkins MR. Reported quality of randomized controlled trials of physiotherapy interventions has improved over time. J Clin Epidemiol. (2011) 64:594–601. 10.1016/j.jclinepi.2010.08.009 21144705

[7] Armijo-Olivo S da Costa BR Cummings GG Ha C Fuentes J Saltaji H. PEDro or Cochrane to assess the quality of clinical trials? A meta-epidemiological study. PLoS ONE. (2015) 10:e0132634. 10.1371/journal.pone.0132634 26161653 PMC4498768

[8] Jadad AR Moore RA Carroll D Jenkinson C Reynolds DJM Gavaghan DJ. Assessing the quality of reports of randomized clinical trials: is blinding necessary? Controll Clin Trials. (1996) 17:1–12. 10.1016/0197-2456(95)00134-4 8721797

[9] Bithell C. Evidence-based physiotherapy: some thoughts on ‘best evidence'. Physiotherapy. (2000) 86:58–9. 10.1016/S0031-9406(05)61206-0

[10] Dijkers MP Murphy SL Krellman J. Evidence-based practice for rehabilitation professionals: concepts and controversies. Arch Phys Med Rehabil. (2012) 93:S164–76. 10.1016/j.apmr.2011.12.014 22683207

[11] Carey TA Stiles WB. Some problems with randomized controlled trials and some viable alternatives. Clin Psychol Psychother. (2016) 23:87–95. 10.1002/cpp.1942 25601435

[12] Krauss A. Why all randomised controlled trials produce biased results. Ann Med. (2018) 50:312–22. 10.1080/07853890.2018.1453233 29616838

[13] van Trijffel E Oostendorp R Elvers JH. Routinely collected data as real-world evidence for physiotherapy practice. Physiother Theory Pract. (2019) 35:805–9. 10.1080/09593985.2019.1615678 31218943

[14] Guyatt G Oxman AD Akl EA Kunz R Vist G Brozek J. GRADE guidelines: 1. Introduction—GRADE evidence profiles and summary of findings tables. J Clin Epidemiol. (2011) 64:383–94. 10.1016/j.jclinepi.2010.04.026 21195583

[15] Djulbegovic B Guyatt GH. Progress in evidence-based medicine: a quarter century on. Lancet. (2017) 390:415–23. 10.1016/S0140-6736(16)31592-6 28215660

[16] Sackett DL Rosenberg WM Gray JM Haynes RB Richardson WS. Evidence based medicine: what it is and what it isn't. BMJ. (1996) 312:71–2. 10.1136/bmj.312.7023.71 8555924 PMC2349778

[17] Tonelli MR. In defense of expert opinion. Acad Med. (1999) 74:1187–92. 10.1097/00001888-199911000-00010 10587679

[18] Gomes-Schwartz B. Effective ingredients in psychotherapy: Prediction of outcome from process variables. J Consult Clin Psychol. (1978) 46:1023. 10.1037/0022-006X.46.5.1023 701541

[19] Krause MS Howard KI. What random assignment does and does not do. J Clin Psychol. (2003) 59:751–66. 10.1002/jclp.10170 12808582

[20] Reed P Osborne LA Whittall CM Emery S. Impact of patient motivation on compliance and outcomes for incontinence. Physiotherapy. (2020). 10.1016/j.physio.2020.10.003 34563914

[21] Osborne LA Whittall CM Edwards DJ Emanuel R Emery S Reed P. Randomised control trial of a values-based motivational interview support to promote attendance at pelvic floor muscle training physiotherapy treatment. J Pelvic Obstetr Gynaecol Physiother. (2016) 119:38–46.

[22] Foldnes N Olsson UH Foss T. The effect of kurtosis on the power of two test statistics in covariance structure analysis. Br J Math Stat Psychol. (2012) 65:1–18. 10.1111/j.2044-8317.2010.02010.x 22233173

[23] Garren ST Osborne KM. Robustness of t-test based on skewness and kurtosis. J Adv Math Comp Sci. (2021) 36:102−110. 10.9734/jamcs/2021/v36i230342

[24] Hartigan SM Dmochowski RR. Gender specific pharmacokinetic and pharmacodynamic considerations for antimuscarinic drugs for overactive bladder treatment. Exp Opin Drug Metab Toxicol. (2020) 16:103–10. 10.1080/17425255.2020.1714591 31918590

[25] Sanetti LM Cook BG Cook L. Treatment fidelity: what it is and why it matters. Learn Disabil Res Pract. (2021) 36:5–11. 10.1111/ldrp.12238

[26] Bakker D Rickard N. Engagement with a cognitive behavioural therapy mobile phone app predicts changes in mental health and wellbeing: MoodMission. Aust Psychol. (2019) 54:245–60. 10.1111/ap.12383

